# Quantitative ToF‐SIMS Assessment of In‐Plane and Out‐of‐Plane Nb Doping Uniformity in CVT‐Grown MoS_2_ Crystals

**DOI:** 10.1002/smtd.202501405

**Published:** 2025-10-20

**Authors:** Itsuki Tanaka, Mian Wei, Tomonori Nishimura, Kaito Kanahashi, Satoru Morito, Keiji Ueno, Amin Azizi, Kosuke Nagashio

**Affiliations:** ^1^ Department of Materials Engineering The University of Tokyo Tokyo 113‐8656 Japan; ^2^ Department of Chemistry Saitama University Saitama 338‐8570 Japan; ^3^ Corporate Research Taiwan Semiconductor Manufacturing Company Ltd. San Jose CA 95134 USA

**Keywords:** 2D materials, chemical vapor transport, molybdenum disulfide, substitutional doping, ToF‐SIMS

## Abstract

Substitutional doping in transition metal dichalcogenides (TMDs) has recently garnered renewed attention due to increasing high demands for ultra‐low contact resistance. However, understanding and controlling the spatial distribution of dopants in 2D TMDs is key for precise modulation of their electronic properties for next‐generation electronics. In this study, chemical vapor transport (CVT) grown Nb‐doped MoS_2_ is used as a model system to quantitatively assess impurity distribution via time‐of‐flight secondary ion mass spectrometry (ToF‐SIMS) and photoluminescence (PL) mapping. In‐plane PL mapping of monolayers reveals spatially uniform Nb incorporation within the lateral resolution limit of ≈1 µm, whereas bulk crystals exhibit variation in Nb content, suggesting limited out‐of‐plane dopant diffusion. Moreover, a non‐destructive method is established for estimating Nb concentration in individual monolayers by leveraging the linear correlation between PL peak position and Nb content, enabling selective use of monolayers with desired doping levels for device applications. The integration of these techniques offers an effective framework for spatially characterizing and fine‐tuning doping in 2D TMDs, paving the way for their reliable implementation into future electronic devices.

## Graphical abstract

By applying ToF‐SIMS to CVT‐grown Nb‐doped MoS2, it is revealed that pronounced inhomogeneity in Nb content exists in the bulk, while monolayers exhibit uniform in‐plane but significant interlayer variation. These observations suggest that out‐of‐plane diffusion of dopants is more restricted than in‐plane diffusion during CVT growth.

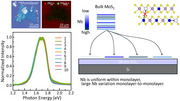

## 1 Introduction

Transition metal dichalcogenides (TMDs) are 2D layered materials composed of a transition metal (e.g., Mo, W) sandwiched between two chalcogen atoms (S, Se, Te). Their strong electrostatic gate control at the monolayer limit in field effect transistors (FETs),^[^ ^1^, ^2^ ^]^ make them highly promising as channel materials for next‐generation logic semiconductor devices. As in conventional Si electronics, substitutional doping is a fundamental technique to control the channel polarity (*n* or *p* type) and to lower the contact resistance.^[^ ^3^ ^]^ However, in TMD‐based FETs, the channel polarity has been mainly tuned via engineering of the metal work function due to their Schottky barrier transistor behavior.^[^ ^4^, ^5^ ^]^ Nonetheless, recent demands for ultra‐low contact resistance^[^ ^6^, ^7^ ^]^ have brought renewed attention to doping strategies. While approaches such as surface charge‐transfer doping^[^ ^8^ ^]^ with chemical species^[^ ^9^, ^10^ ^]^ or oxides^[^ ^11^, ^12^, ^13^ ^]^ have been explored, substitutional doping at the transition metal site, compared to that at the chalcogen site,^[^ ^14^, ^15^ ^]^ remains the most robust method, particularly in terms of thermal stability.^[^ ^16^, ^17^ ^]^

**Figure 1:**
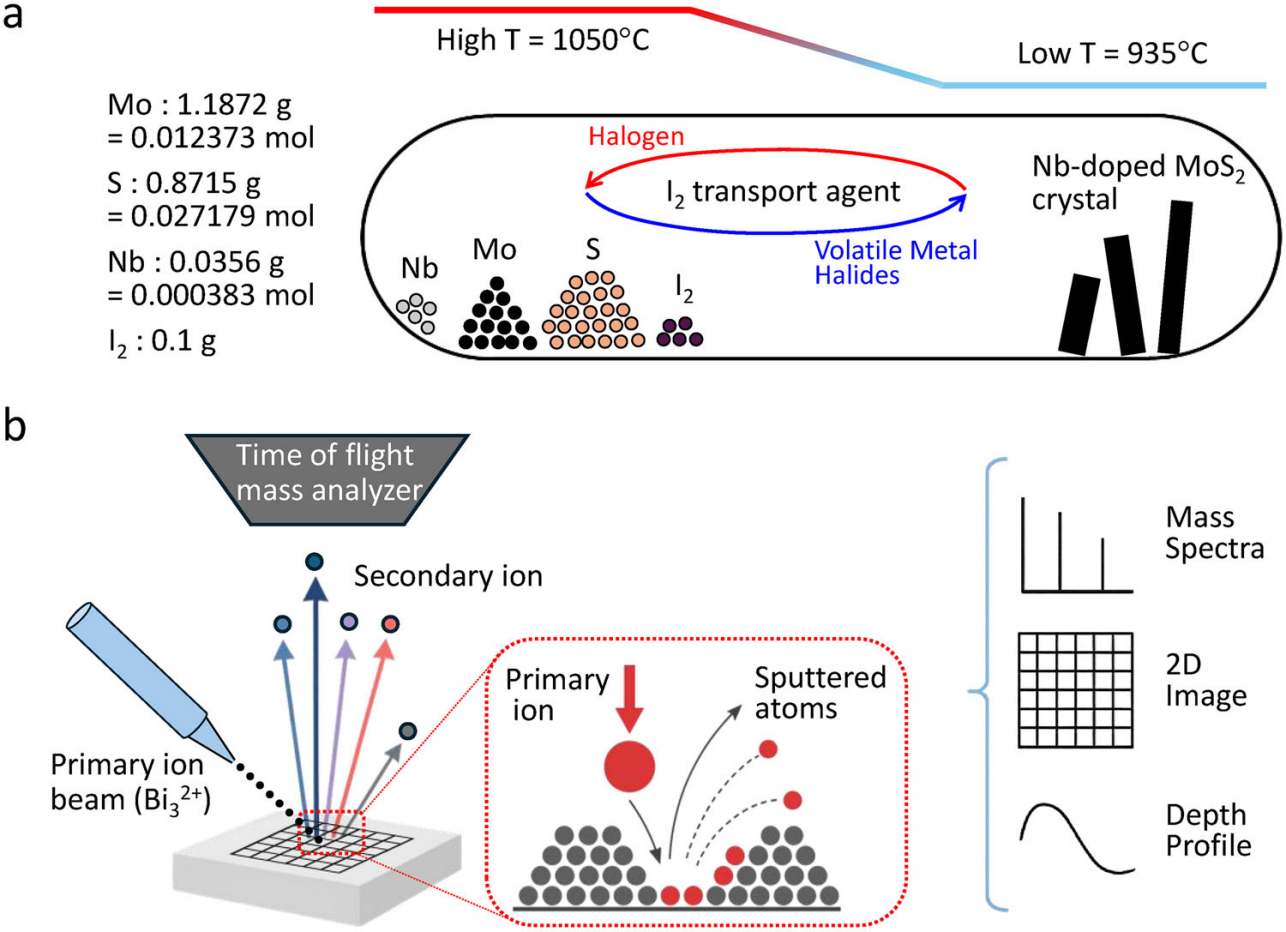
a) Schematic illustration of CVT growth. The dopant ratio introduced during growth is assumed to match the composition of the resulting crystal (nominal composition). b) Schematic illustration of ToF‐SIMS. A primary ion beam (e.g., Bi_3_^2+^) sputters the sample surface, ionizing the ejected species, which are then detected by a time‐of‐flight mass analyzer. By raster‐scanning the primary ion beam, one can generate 2D ion maps, and by alternating these measurements with Ar^+^ sputtering, depth profiles can be obtained.

Nb has long been employed as a *p*‐type substitutional dopant in TMDs, notably in Nb‐doped MoS_2_^[^ ^18^ ^]^ and WSe_2_^[^ ^19^, ^20^ ^]^ synthesized via chemical vapor transport (CVT), as shown in **Figure** **1**a. A nominal Nb substitution of 0.5% in bulk MoS_2_, commercially available, has been shown to convert its intrinsic *n*‐type behavior to *p^+^*‐type conductivity.^[^ ^18^ ^]^ Hall measurements have confirmed a degenerate carrier concentration on the order of 2 × 10^19^ cm^−3^. However, photoluminescence (PL) measurements from monolayer flakes exfoliated from bulk Nb‐doped MoS_2_ crystals reveal variability in PL peak positions (Figure S1, Supporting Information), suggesting possible spatial inhomogeneity in Nb incorporation. The observed non‐uniformity may arise from the specific growth conditions used for the crystals in this work, highlighting the importance of optimizing synthesis for consistent doping and thorough characterization of dopant distribution. Additionally, systematic quantification of the dopant concentration and distribution in monolayer TMDs (e.g., MoS_2_) has not been fully realized due to the inherent difficulties in characterizing monolayer samples with a thickness of only 0.65 nm and lateral dimensions of typically ≈100 µm^2^. While high‐resolution techniques such as high‐angle annular dark‐field scanning transmission electron microscopy (HAADF‐STEM) can provide detailed insights, their field of view is typically limited to only several tens of nanometers.^[^ ^21^, ^22^ ^]^ Moreover, distinguishing Nb atoms from Mo atoms in Nb‐doped MoS_2_ remains challenging due to their adjacent atomic numbers. In contrast, time‐of‐flight secondary ion mass spectrometry (ToF‐SIMS)^[^ ^23^ ^]^ offers excellent surface sensitivity and spatial resolution on the order of a few µm^2^, since it employs a low‐dose primary ion beam (≈10^12^ cm^−2^) of heavy ions or clusters such as Bi_3_^2+^ to gently sputter the sample surface, as shown in Figure 1b. These characteristics make ToF‐SIMS particularly well‐suited for investigating dopant uniformity in monolayer TMDs. However, quantitative analysis using ToF‐SIMS requires calibration against standard samples with known dopant concentrations to determine relative sensitivity factors (RSFs) for accurate quantification.

To date, ToF‐SIMS has been employed to detect organic contaminants on exfoliated MoS_2_ surface^[^ ^24^ ^]^ and to characterize impurities in natural MoS_2_ crystals qualitatively.^[^ ^25^ ^]^ While quantitative ToF‐SIMS analyses of dopants in TMDs using standard samples remain limited, Kozhakhmetov et al. recently quantified Re concentrations in wafer scale Re‐doped WSe_2_ grown by metal‐organic chemical vapor deposition (MOCVD), achieving a detection limit of ≈0.07% by calibrating the RSF with X‐ray photoelectron spectroscopy (XPS) data from large‐area samples.^[^ ^26^ ^]^ However, their study did not examine dopant spatial uniformity and relied on the availability of large‐area TMDs. In contrast, the quantitative analysis of monolayers exfoliated from CVT‐grown crystal remains challenging due to the limited lateral dimensions of the flakes.

In this work, we apply ToF‐SIMS with a primary Bi_3_^2+^ ion beam to quantitatively evaluate the Nb concentration in Nb‐doped MoS_2_ flakes exfoliated from CVT‐grown crystals, employing standard samples calibrated by XPS. To ensure sufficient ion counts while maintaining static ToF‐SIMS conditions, relatively large‐area Nb‐doped MoS_2_ monolayers were prepared via a Au‐mediated exfoliation technique (Figure S2, Supporting Information).^[^ ^27^ ^]^ This approach enabled accurate determination of the [Nb]/([Mo] + [Nb]) atomic ratio even in monolayer flakes, an important advancement in overcoming the analytical challenges associated with such ultrathin structures. Our analysis reveals that the in‐plane distribution of Nb is spatially uniform in monolayer MoS_2_, while out‐of‐plane variability was found in the bulk crystal. In particular, some variations in the Nb concentration of monolayer flakes exfoliated from the same bulk crystal were observed. Recognizing the inherently destructive nature of ToF‐SIMS, we also propose a complementary, non‐destructive approach based on PL measurements to estimate Nb concentrations in Nb‐doped MoS_2_ monolayers. We observe that increasing Nb concentrations induce a monotonic redshift in the Nb‐bound exciton emission peak, thereby establishing a non‐destructive technique for doping level estimation.

## 2 Results and Discussion

Nb‐doped MoS_2_ bulk crystals were grown by CVT with various Mo:Nb powder source ratios, as shown in Figure S3 (Supporting Information). Importantly, the nominal Nb concentration, which was determined by the precursor ratio during CVT growth, does not necessarily reflect the actual Nb content in the MoS_2_ crystals. Hereafter, Nb‐doped MoS_2_ is expressed simply as Nb‐MoS_2_.

**Figure 2:**
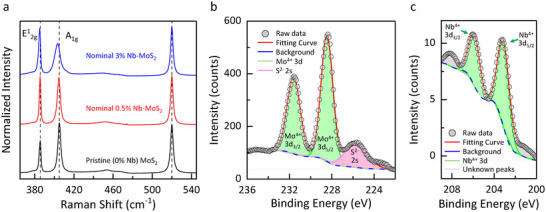
a) Raman spectra of pristine and Nb‐doped MoS_2_ monolayers exfoliated from CVT‐grown crystals via a Au‐mediated transfer technique. The intensity is normalized to the Si peak at 520.02 cm^−1^. b) Mo 3d and c) Nb 3d XPS spectra obtained from Nb‐MoS_2_ flakes transferred on a Si/SiO_2_ substrate. Binding energies were calibrated by referencing the Si^4+^ 2p peak of the SiO_2_ substrate at 103.9 eV.

First, monolayer Nb‐MoS_2_ exfoliated from CVT‐grown crystal with different nominal Nb concentrations was characterized by Raman spectroscopy, as shown in **Figure** **2**a. For all samples of MoS_2_ (pristine, nominal 0.5%, and nominal 3%), the peak separation between A_1g_ and E^1^_2g_ was lower than 20 cm^−1^, confirming that the flakes were monolayers.^[^ ^28^ ^]^ Comparing the nominal 3% sample with the pristine one, only the A_1g_ peak exhibited softening and broadening. The increase in Nb substitution seems to increase sulfur vacancies (V_S_). While Nb acts as an acceptor in MoS_2_, V_S_ serves as electron donors.^[^ ^29^ ^]^ Moreover, the high ionization energy of Nb in monolayer MoS_2_ (≈400 meV)^[^ ^21^, ^30^ ^]^ hinders effective hole generation. Consequently, an increase in electron density upon Nb substitution might be expected, as shown by Raman change in Nb‐MoS_2_ in previous studies.^[^ ^18^ ^]^ The observed softening and broadening of the A_1g_ peak can thus be attributed to enhanced electron‐phonon coupling, providing spectroscopic evidence of increased electron density.^[^ ^31^ ^]^ In addition, the E^1^_2g_ peak remains essentially unchanged, indicating that strain from substitutional doping is minimal compared to that from the transfer process.^[^ ^32^ ^]^ The nominal 0.5% sample exhibits the same systematic Raman trends as the nominal 3% sample, albeit to a smaller extent, consistent with its lower Nb content.

While the Raman shifts suggest the presence of Nb doping, quantifying the substitution level solely from Raman data is challenging. For quantitative analysis of Nb concentration using ToF‐SIMS, it is necessary to predetermine the [Nb]/([Mo]+[Nb]) ratio by an independent method. Due to the limited sensitivity of XPS, nominal 3% Nb‐MoS_2_ was selected. Figure 2b,c shows Mo 3d, S 2s, and Nb 3d XPS peaks from Nb‐MoS_2_ flakes, respectively. The Mo^4+^ 3d peaks have lower binding energy than that of pristine MoS_2_, indicating degenerate *p*‐type doping.^[^ ^18^ ^]^ Nb^4+^ 3d_5/2_ and Nb^4+^ 3d_3/2_ peaks appeared at 203.1 and 205.9 eV, respectively, which is in agreement with the previous report.^[^ ^18^ ^]^ Based on the area ratio of Mo and Nb peaks, the Nb concentration was estimated to be 1.8%, which represents an average concentration across multiple flakes, given the ≈1 mm^2^ spatial resolution of XPS. Notably, although the sample used for XPS measurements was prepared with a nominal Nb concentration of 3%, only 1.8% Nb was incorporated into MoS_2_ crystals. This deviation between the nominal and actual dopant concentrations is consistent with a previous report on CVT‐grown Nb‐doped MoSe_2_ and WSe_2_.^[^ ^33^ ^]^

**Figure 3:**
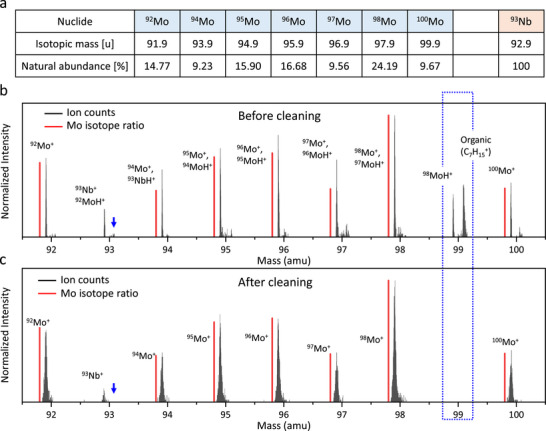
a) Isotopic abundance ratios of Mo and Nb. b) ToF‐SIMS mass spectrum around the Mo and Nb region before Ar^+^ sputtering. Organic peaks, caused by transfer‐related contamination, appear at masses slightly above integer values. MoH^+^ peaks also appeared, overlapping with the Nb^+^ peak. The red bars represent the ideal Mo isotopic ratio, which is intentionally shifted toward the lower mass side for clarity. c) ToF‐SIMS mass spectrum around the Mo and Nb region after Ar^+^ sputtering. The Mo^+^ peak intensities match the natural isotopic ratio, indicating that the MoH^+^ peak has been removed.

To quantitatively evaluate the Nb dopant concentration within individual transferred flakes, ToF‐SIMS measurements were conducted. **Figure** **3**a displays the seven stable isotopes of Mo, the single isotope of Nb, and their corresponding natural abundance ratios.^[^ ^34^ ^]^ Although ToF‐SIMS data were acquired across the entire mass spectrum, as shown in Figure S4 (Supporting Information), the analysis was primarily focused on the 92–100 amu range. Importantly, Mo does not possess an isotope at mass 93, which allows for a clear distinction between Mo^+^ and Nb^+^ signals.

Figure 3b shows the mass spectra acquired from one of the Nb‐MoS_2_ bulk flakes previously analyzed by XPS, without any surface cleaning. In this spectrum, both inorganic and organic peaks are evident. Although Nb does not have an isotope at 99 amu, strong peaks were detected at this position, as shown by the blue dotted box, indicating that signals at 93 amu are also likely influenced by the combination of Nb‐related ions and surface contaminants. As commonly observed in tape‐exfoliated samples, organic residues remain on the surfaces of both the TMD flakes and the SiO_2_/Si substrate.^[^ ^24^ ^]^ The high mass resolution of ToF‐SIMS typically distinguishes between inorganic ions, which appear slightly below their nominal mass values, and organic ions, that appear slightly above them.^[^ ^23^ ^]^ However, the presence of these residues can lead to the formation of hydrogenated metal ions. For example, hydrogenated molybdenum (^92^MoH⁺) appears at 92.915 amu and completely overlaps with the ^93^Nb⁺ signal at 92.906 amu.^[^ ^34^ ^]^ This overlap makes it difficult to distinguish the two species even with the high mass resolution of ToF‐SIMS, and necessitates the reduction of hydrogenated interferences to accurately quantify ^93^Nb⁺ relative to ^92^MoH^+^.

In previous study on MOCVD‐grown Re‐WSe_2_,^[^ ^26^ ^]^ a system also exhibiting mass overlaps due to adjacent atomic weights, low levels of organic contamination enabled the correction of hydrogenated components based on known isotopic ratios, permitting precise Re quantification. In contrast, the presence of significant organic contamination introduced during the Au‐mediated transfer process inhibited reliable Nb quantification without surface cleaning. To address this, a brief 10‐second Ar^+^ sputtering was performed prior to measurement. As shown in Figure 3c, this treatment eliminated the ^98^MoH⁺ peak at 99 amu, and other peaks shifted above nominal values also disappeared (as shown by the blue arrow). These results confirm effective removal of both organic residues and hydrogenated species, allowing the isolation of intrinsic Mo^+^ and Nb^+^ signals. The resulting isotopic peak intensities aligned with natural abundance ratios, enabling determination of the relative elemental composition from the [Nb^+^]/([Mo^+^] + [Nb^+^]) signal ratio. Although V_S_ may be created due to the Au‐mediated transfer, the Nb concentration can still be accurately determined, as it is evaluated based on the [Nb]/([Mo] + [Nb]) atomic ratio.

**Figure 4:**
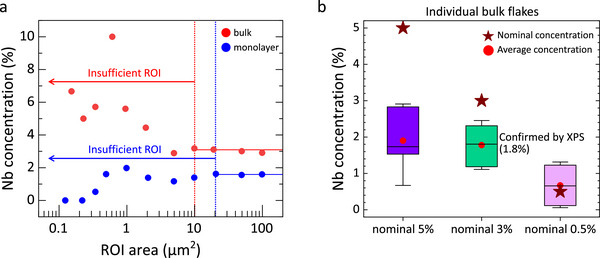
a) Nb concentration in nominal 3% Nb‐MoS_2_ bulk and monolayer flakes plotted as a function of ROI area in ToF‐SIMS. Since the Nb signal intensity scales linearly with the ROI area, the calculated Nb concentration exhibits significant fluctuations when the ROI is too small. b) Flake‐by‐flake variation in Nb concentration for Nb‐MoS_2_ bulk flakes exfoliated from three different crystals with different nominal Nb concentrations.

Next, the relationships between the analyzed areas and the accuracy of impurity quantification are discussed. As the crystal size increases, a larger region of interest (ROI) becomes available for analysis, leading to improved counting statistics. **Figure** **4**a plots the calculated Nb concentration as a function of ROI size, obtained from both bulk and monolayer Nb‐MoS_2_ crystals using ToF‐SIMS. When the ROI becomes smaller than 10 µm^2^, significant fluctuations appear in the calculated Nb concentration, indicating that an accuracy of ±0.1% cannot be reliably achieved in such small areas. In this study, we target an accuracy of ±0.1% in Nb concentration. A measurement area of 100 µm^2^, which is smaller than a typical exfoliated flake, is sufficient to meet this criterion. Therefore, throughout this work, the ROI was fixed at 100 µm^2^, and a measurement precision of ±0.1% is assumed. This ROI‐determined accuracy of impurity quantification also defines the detection limit. For reference, Kozhakhmetov et al. achieved a detection limit of ≈0.07% for Re‐WSe_2_ using ToF‐SIMS.^[^ ^26^ ^]^ Their enhanced sensitivity resulted from analyzing a wafer‐scale WSe_2_ film grown by MOCVD, which enabled the use of an ROI as large as 10 000 µm^2^. Since statistical uncertainty scales with the inverse square root of the total counts, increasing the area by a factor of 100 provides 100 times more counts, thereby improving the precision to ≈±0.01%. This high precision allowed them to achieve a detection limit of 0.07%. In contrast, due to the limited lateral size (≈100 µm^2^) of our exfoliated flakes, the practical detection limit in our case is ≈0.1–0.2%. However, it is worth noting that quantifying the concentrations of V_Mo_ or V_S_, which are typically ≈0.1% in high quality monolayers,^[^ ^35^, ^36^ ^]^ is challenging, as it requires ion intensity measurements with a relative precision of ≈10^−4^.

ToF‐SIMS measurements were subsequently performed on multiple individual bulk flakes with a nominal 3% Nb‐MoS_2_ (corresponding to 1.8% Nb by XPS). These measurements revealed variation in the [Nb^+^]/([Nb^+^] +  [Mo^+^]) count ratio among flakes. It is important to note that the XPS data, which average over an area of ≈1 mm^2^, represents the mean Nb concentration of multiple flakes, each a few hundred µm^2^ in size. By correlating the average ToF‐SIMS count ratio with the 1.8% Nb concentration determined by XPS for the nominal 3% samples, the RSF was derived for Nb quantification in MoS_2_. Figure 4b presents the distribution of measured Nb concentrations for individual bulk flakes with various nominal Nb concentration, based on the RSF. In ToF‐SIMS measurements at high Nb concentrations like Mo_0.5_Nb_0.5_S_2_ alloy, saturation of the secondary ion yield and signal variations caused by clustering, known as the matrix effect, can lead to a loss of RSF linearity, making simple extrapolation potentially inaccurate. However, since the concentration range investigated in this study is limited, the RSF linearity is expected to be preserved. At the highest nominal Nb concentration (5%), both the discrepancy between nominal and actual concentrations and the flake‐to‐flake variability are most pronounced. The difference between the highest and lowest measured Nb concentrations reaches 2.2%. Importantly, even in commercially available Nb‐MoS_2_ samples nominally doped at 0.5%, widely used in previous reports,^[^ ^29^, ^30^, ^37^, ^38^, ^39^, ^40^ ^]^ substantial variability was observed, with some flakes exhibiting Nb concentrations as low as 0.06%. However, considering the detection limit, the actual concentration in these flakes is likely >0.1%. The variations in dopant concentrations across different flakes may stem from the growth conditions used in this study. This highlights the importance of thoroughly assessing the dopant concentration in flakes prior to device integration, as variation in dopant concentrations can result in considerable performance variability.

Interestingly, the actual Nb concentration in the nominal 5% sample was only 1.9%, similar to that of the nominal 3% sample. Furthermore, one flake from the nominal 5% batch exhibited an actual Nb concentration of just 0.7%, which is lower than that of any flake in the nominal 3% batch. These unexpected trends can be understood by examining optical images of the grown flakes: the lateral flake size generally decreases with increasing nominal Nb concentration, but then increases again at the 5% level. The recovery in size suggests a reduced actual Nb incorporation in the 5% sample compared to the 3% sample. Moreover, we observed that the flake brittleness increases with higher actual Nb content.

By calibrating the ToF‐SIMS intensity ratio with the average Nb concentration obtained via XPS, we established the RSF, which was then applied to estimate Nb concentrations in other Nb‐MoS_2_ samples where XPS measurements were not feasible, as shown in Figure S3a (Supporting Information). To avoid ambiguity and ensure clarity in subsequent analyses, we will refer to the actual Nb concentration as the basis for all experimental analyses and interpretations.

**Figure 5:**
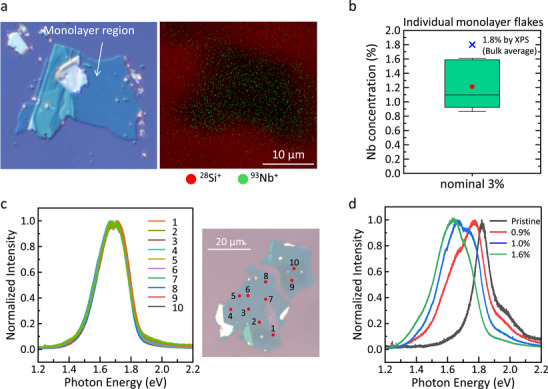
a) Optical microscope image of the monolayer Nb‐MoS_2_ flake and ToF‐SIMS elemental mapping of the same region for ^28^Si^+^ and ^93^Nb^+^. Monolayer flake was transferred from a 1.8% Nb‐MoS_2_ crystal. b) Flake‐by‐flake variation in Nb concentration for various Nb‐MoS_2_ monolayer flakes transferred from a 1.8% Nb‐MoS_2_ crystal. c) In‐plane PL mapping of a single Nb‐MoS_2_ monolayer flake. The PL intensities are normalized. Measurements at ten different locations yielded identical normalized PL spectra, indicating that the Nb concentration is uniform across the flake. d) PL spectra of pristine MoS_2_ and Nb‐MoS_2_ monolayer flakes, where Nb concentrations were quantitatively determined by ToF‐SIMS.

To investigate the lateral distribution of Nb in monolayer flakes, we performed ToF‐SIMS measurements on monolayer flakes exfoliated from a 1.8%‐Nb‐MoS_2_ bulk crystal after Ar^+^ cleaning. As shown in **Figure** **5**a, the Nb signal distribution corresponded well with the flake morphology, confirming the validity of the measurement. The relationship between the actual Nb concentration and ROI was again analyzed, as shown in Figure 4a. For ROIs smaller than 20 µm^2^, intrinsic Nb signals were obscured by measurement noise due to the inherently low ion counts of the monolayer, thereby hindering accurate quantification. Accordingly, a maximum ROI of 100 µm^2^ was selected, within which the Nb concentration in the monolayer was evaluated to be 1.6%. This represents the first quantification of dopant concentration in transferred monolayer flakes, demonstrating that ToF‐SIMS can serve as an effective post‐fabrication technique for evaluating dopant levels in monolayer samples with lateral dimensions exceeding 100 µm^2^. Similar measurements using 100 µm^2^ ROIs were performed on additional monolayer flakes exfoliated and transferred from the same 1.8% Nb‐doped bulk crystal, as shown in Figure 5b. As expected from the variation of the Nb concentration in the bulk crystals (Figure 4b), these monolayers exhibited corresponding variations in Nb concentration.

Given the insufficient lateral size of the current monolayers for mapping spatial dopant inhomogeneity via ToF‐SIMS, PL mapping was employed as an alternative approach. Using a 488 nm excitation laser and a 1 µm^2^ illumination area, PL spectra were collected from ten distinct positions on a large‐area Nb‐doped MoS_2_ monolayer, as shown in Figure 5c. The obtained PL peaks were identical regardless of measurement positions, suggesting that Nb concentration is laterally uniform within each monolayer, though it varied from flake to flake. This laterally uniform PL data indicates that a single‐point PL spectrum of monolayer represents the whole area, which can be correlated with Nb concentration determined via ToF‐SIMS measurements. Figure 5d presents PL spectra obtained from different monolayer flakes with various Nb concentration. As observed, the PL peak exhibits a redshift as the Nb concentration increases, consistent with previous reports on Nb‐MoS_2_.^[^ ^30^ ^]^ This redshift, which can be as large as ≈0.2 eV, is substantially larger than the tens of meV redshifts typically arising from band gap modifications due to strain or from changes in the exciton‐trion ratio induced by V_S_ formation, both of which may be introduced by the Au transfer method (Figure S5, Supporting Information). It should be emphasized that monolayer flakes with Nb concentrations in the 1.0–1.6% range were obtained from the same bulk crystal with an actual Nb content of 1.8%. This PL variability motivated the present study, as outlined in the introduction. Notably, in MoSe_2_ and WS_2_, Nb doping renders nonradiative exciton recombination dominant, leading to a pronounced reduction in PL intensity.^[^ ^41^, ^42^ ^]^ In contrast, in Nb‐MoS_2_, efficient radiative recombination of neutral acceptor bound excitons (A^0^X) largely preserves the PL intensity in lower energy.^[^ ^30^ ^]^ This relatively high PL intensity allowed us to recognize the fluctuation in Nb concentration.

**Figure 6:**
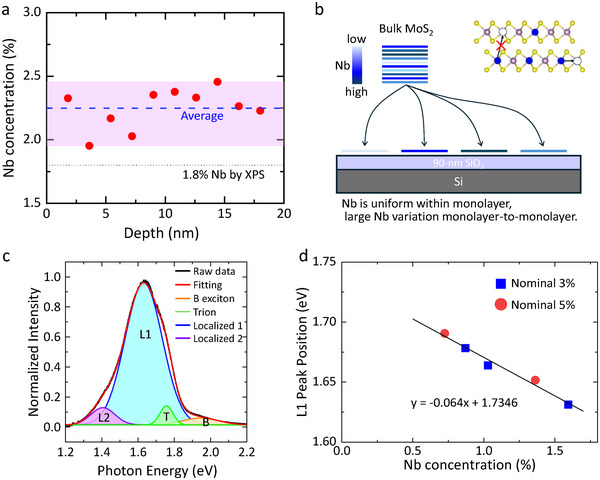
a) Dopant depth distribution in the 1.8%‐Nb‐MoS_2_ bulk crystal obtained using ToF‐SIMS. The dotted line represents the average Nb concentration of 2.25%. b) Schematic illustration of Nb‐MoS_2_ bulk crystal grown by CVT. c) PL peak deconvolution of a 1.6% Nb‐MoS_2_ PL spectrum, fitted using Gaussian functions. d) Relationship between the Nb‐bound exciton peak position and Nb concentration, showing a linear reduction in the PL emission energy with increasing Nb concentration.

Next, we investigated the depth dopant distribution in the 1.8%‐Nb‐MoS_2_ bulk crystal using ToF‐SIMS, applying alternating cycles of Ar⁺ sputtering and Bi_3_^2+^ analysis. The sputtering rate was estimated to ≈1.8 nm per minute based on laser profilometry measurements of crater depth, as shown in Figure S6 (Supporting Information). The depth profile in **Figure** **6**a reveals clear vertical inhomogeneity with the Nb concentration varying by up to 0.5% around an average of 2.25%.

Combined ToF‐SIMS and PL mapping analyses (Figures 5c and 6a) suggest that while the Nb concentration within individual monolayers is laterally uniform, the vertical distribution across the bulk crystal is non‐uniform, as shown in Figure 6b. In general, ion mixing during sputtering tends to smooth out compositional variations under certain conditions.^[^ ^43^ ^]^ Therefore, the fact that vertical inhomogeneity was observed despite such mixing suggests that it may originate from the specific growth conditions of crystal growth process. A possible mechanism might be that, since CVT growth employs powder sources, achieving precise control over the precursor flux might be challenging due to changes in their surface area over time, which may lead to compositional variations within the bulk crystal. Moreover, the vertical diffusion of Nb may be limited by the interlayer van der Waals gaps, whereas in‐plane diffusion could be enhanced at the growth temperature of 935 °C through vacancy‐assisted pathways. Although no quantitative information on the vertical diffusion constant during CVT is available, it is worth noting that when monolayer MoS_2_ was epitaxially grown on monolayer WS_2_ at 850 °C,^[^ ^44^ ^]^ no atomic migration was observed even though WS_2_‐MoS_2_ system is a complete solid solution.^[^ ^45^ ^]^ While epitaxial growth conditions differ fundamentally from those of CVT, where all precursors of Nb, Mo, and S are supplied, this example nevertheless suggests that out‐of‐plane diffusion of transition‐metal atoms may be inherently limited. Therefore, Nb‐MoS_2_ bulk crystals may exhibit vertical inhomogeneity but maintain lateral uniformity, with the monolayers exfoliated from them showing lateral uniformity in Nb concentration.

Due to the observed dopant inhomogeneity in CVT‐grown Nb‐MoS_2_ bulk crystals, it is crucial to identify MoS_2_ monolayers with appropriate Nb doping levels for desired device application. Therefore, we examined the correlation between Nb concentration and the PL spectral features. Figure 6c presents a representative PL spectrum with peak separation of a monolayer MoS_2_ with 1.6% Nb doping. The A exciton peak, typically located at ≈1.85 eV in pristine monolayer MoS_2_,^[^ ^46^ ^]^ is absent. Instead, two redshifted peaks appear at ≈1.65 eV (L1) and ≈1.40 eV (L2). Notably, the position of the L1 peak exhibits a linear relationship with Nb concentration, as shown in Figure 6d, suggesting that this peak is related to Nb and is likely associated with a Nb‐bound exciton as proposed in the previous literature.^[^ ^30^ ^]^ The fundamental mechanism underlying the redshift of the L1 peak with increasing Nb concentration can be explained by the band gap reduction with increasing impurity concentration.

However, Nb doping may also promote the formation of V_S_ and Nb‐V_S_ complexes near transition metal sites,^[^ ^21^, ^47^ ^]^ which could serve as alternative luminescence centers. The L2 peak is likely associated with these defect‐induced states. To elucidate this, it will be necessary to separate the contribution of Nb concentration from those of increased V_S_ and carrier density modulations. Strategies such as annealing in a sulfur‐rich environment to reduce V_S_ concentration, and modulating carrier density via gate bias^[^ ^48^, ^49^, ^50^ ^]^ will be critical for this evaluation. Nonetheless, the observed linear relationship between the L1 peak position and Nb concentration indicates that PL spectroscopy serves as an effective and non‐destructive tool for estimating Nb concentrations in monolayer MoS_2_ exfoliated from CVT‐grown crystals. It should be noted, however, that the present calibration curve cannot be directly applied to CVD‐grown Nb‐MoS_2_,^[^ ^50^, ^51^, ^52^ ^]^ as it includes PL shifts induced by strain.

Finally, we discuss the CVT growth of Nb‐doped MoS_2_. While I_2_ is a suitable transport agent for the CVT growth of MoS_2_ single crystals,^[^ ^53^ ^]^ the introduction of Nb, even at a nominal 1%, significantly suppresses crystal growth, resulting in a noticeable reduction in both the yield and size of the obtained crystals, as shown in Figure S3b (Supporting Information). As the nominal Nb concentration increases, the degree of growth suppression becomes more pronounced. Although the growth durations were extended more, no further crystal growth was observed. Instead, an increased occurrence of needle‐like crystals was noted among the resultant products. Raman and XPS analysis confirmed that these needle‐like structures are composed of Nb_2_O_5_. Consequently, as observed in the nominal 5% Nb‐doped samples, the actual Nb concentration incorporated into MoS_2_ decreases to 1.9%. At a nominal doping level of 10% (not shown), the formation of Nb‐doped MoS_2_ crystals is entirely inhibited. Although CVT is often regarded as a thermodynamically equilibrium process, practical factors such as the gradual change in the surface area of the source powders over time can affect transport dynamics. Consequently, the concentration of the transporting species in the gas phase may fluctuate over the multi‐day growth period, depending on the timing of crystal nucleation events, as shown in Figure S3c (Supporting Information). Nevertheless, CVT remains the most reliable technique for obtaining substitutionally doped bulk crystals, as compared to alternative approaches (Figure S7, Supporting Information). Therefore, careful optimization of CVT growth conditions, along with detailed characterization, is essential to achieve controlled dopant concentrations and uniform dopant distribution.

## 3 Conclusion and Future Outlook

In this work, we employed ToF‐SIMS and prior surface treatments to successfully quantify Nb concentrations in MoS_2_ flakes with limited lateral dimensions. Detailed analysis of bulk crystals suggested spatial inhomogeneity in Nb concentration, whereas monolayer flakes exhibited uniform in‐plane distribution. In addition, we demonstrated how PL spectroscopy can be used as an effective and non‐destructive technique to estimate dopant concentrations in monolayer TMDs. These combined techniques provide a powerful toolkit for spatially characterizing, understanding, and optimizing doping in 2D TMDs, which is essential for their reliable integration into next‐generation electronic devices.

## 4 Experimental Section

### Sample Preparation

The mixture of Mo, Nb, and S powders and I_2_ pieces was loaded into a quartz ampule inside an Ar‐filled glovebox. I_2_ was used as a transport agent. The ampule was then evacuated to ≈10^−4^ Pa and sealed using a gas burner. To reduce the number of crystal nuclei, the system was first maintained under a reversed temperature gradient (growth side: 1050 °C, source side: 935 °C) for three days, shifting some of the source material toward the growth side. The temperature gradient was then reversed to the forward direction (growth side: 935 °C, source side: 1050 °C) for seven days to promote crystal growth of Nb‐doped MoS_2_. Bulk TMD crystals were exfoliated and coated with ≈50 nm of Au. The Au‐coated flakes were picked up with a polyurethane film and transferred onto a SiO_2_/Si substrate. The film was removed by immersion in butyl acetate for 5 min, and Au was etched in a KI/I_2_ solution. Finally, the substrate was rinsed with deionized water and dried under N_2_, yielding large‐area monolayers.

### Measurement

Raman and PL measurements were carried out at room temperature using a HORIBA LabRAM HR‐800 spectrometer with a 488 nm excitation laser. The laser power was set to 45 µW for Raman and reduced to 4.5 µW for PL measurements to prevent sample damage and minimize heating effects. XPS measurements were performed under ultra‐high vacuum conditions with a base pressure of 1 × 10^−7^ Pa using a JEOL JPS‐9010 system equipped with a Mg Kα X‐ray source. ToF‐SIMS measurements were carried out using a ULVAC‐PHI TRIFT V nanoTOF instrument. A 30 keV Bi_3_^2+^ cluster primary ion beam was raster‐scanned across the sample surface. Positive secondary ion spectra were acquired with high sensitivity for Nb^+^ and Mo^+^ signals, over a mass range of 0–380 amu. No electron or ion charge compensation was applied during measurements. Each image was acquired with a resolution of 256 × 256 pixels. The specimen stage was biased at +3.75 kV, and the contrast diaphragm was kept fully open with no additional masks or grids used during analysis. The analysis chamber pressure was maintained in the low 10^−6^ Pa range throughout the experiments.

## Conflict of Interest

The authors declare no conflict of interest.

## Supporting information



Supporting Information

## Data Availability

The data that support the findings of this study are available from the corresponding author upon reasonable request.
