# Supplementary material for: Quantitative ToF‐SIMS Assessment of In‐Plane and Out‐of‐Plane Nb Doping Uniformity in CVT‐Grown MoS2 Crystals
Source: Small Methods. 2025 Oct 20;9(12):e01405. doi: 10.1002/smtd.202501405 (PMC12716208; doi:10.1002/smtd.202501405)
Supplement: Supplementary file 1 — Supporting Information [file SMTD-9-e01405-s001.pdf]

Copyright WILEY-VCH Verlag GmbH & Co. KGaA, 69469 Weinheim, Germany, 2018.
Supporting Information

Quantitative ToF-SIMS Assessment of In-Plane and Out-of-Plane Nb Doping Uniformity in CVT-Grown MoS_2 Crystals

*Itsuki Tanaka, Mian Wei, Tomonori Nishimura, Kaito Kanahashi, Satoru Morito, Keiji Ueno, Amin Azizi, and Kosuke Nagashio**

S1. PL measurements of commercially available Nb-doped MoS_2

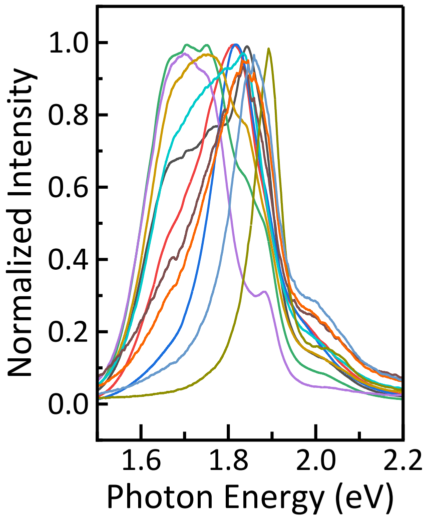

Figure S1. PL results of commercially available Nb-doped monolayer MoS_2 (nominal 0.5% Nb) measured at room temperature. Large variations were observed.

S2. Au-mediated transfer method

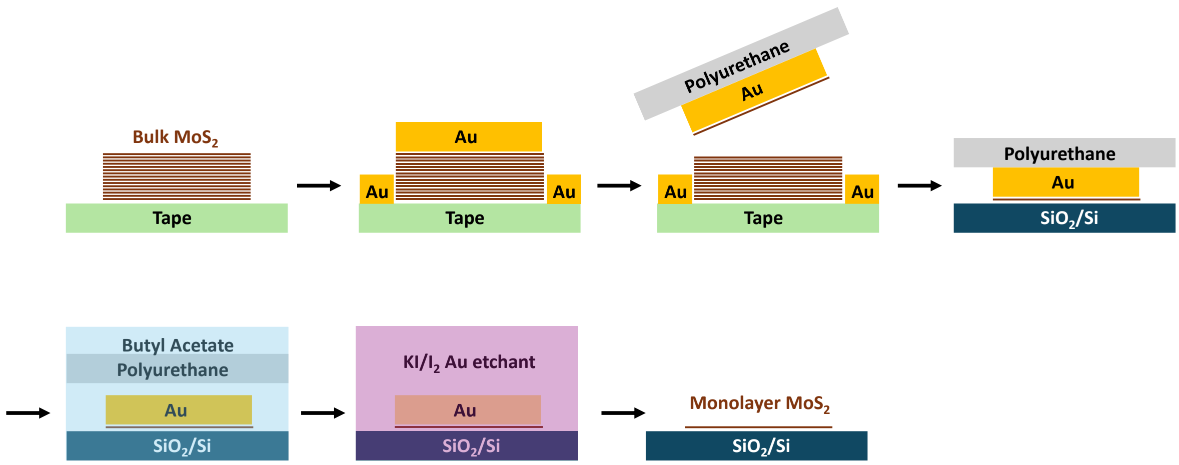

Figure S2. Schematic illustration of the Au-mediated transfer method.

Note: Bulk MoS_2 crystals were first mechanically exfoliated using adhesive tape following a standard procedure. Subsequently, a ~50 nm thick layer of Au was deposited onto the surface of the MoS_2 crystal while it remained on the tape. The MoS_2 crystal along with the deposited Au layer was then lifted off using a polyurethane (PU) film. Because the thiol bonds between Au and S were stronger than van der Waals forces, the monolayer readily detached. Next, the resulting PU/Au/monolayer-MoS_2 stack was transferred onto a SiO_2/Si substrate. The Au layer was removed using a KI/I_2 etchant. The sample was then thoroughly rinsed with deionized water and dried using a nitrogen gas flow, yielding a large-area monolayer MoS_2 on the SiO_2/Si substrate.

S3. Information about Nb-doped MoS_2 crystals grown by CVT

a

Sample	Nominal Nb concentration	effective Nb concentration	
Pristine	0	0	
1	0.5%	Ave 0.4%	ToF-SIMS
2	3%	Ave 1.8%	XPS standard
3	5%	Ave 1.9%	ToF-SIMS

b

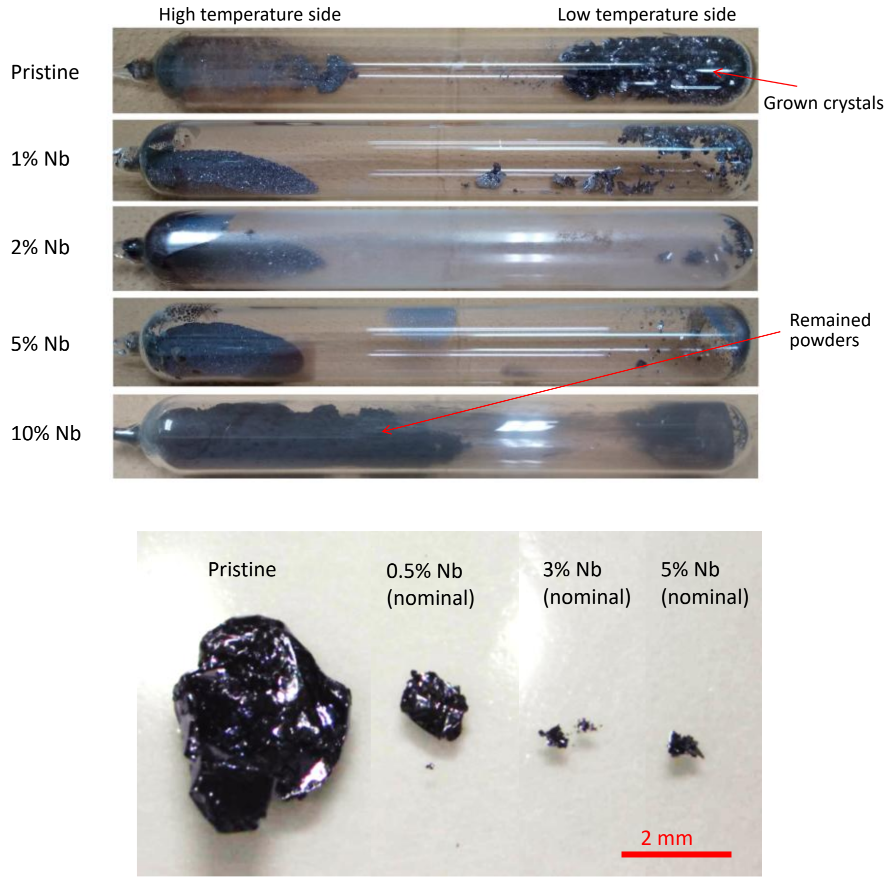

c

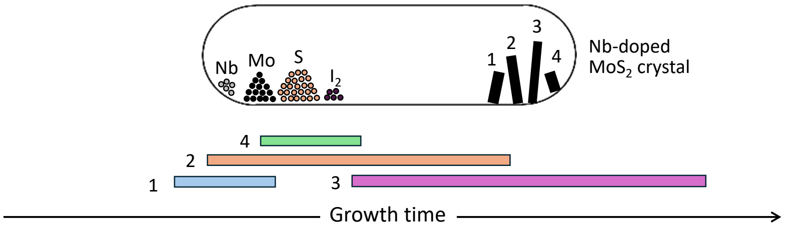

Figure S3. a) Nb concentration data for Nb-doped MoS_2 crystals grown by CVT. The *nominal Nb concentration* refers to the initial proportion of Nb powder introduced prior to the growth process, whereas the *actual Nb concentration* was quantified via ToF-SIMS, calibrated against a standard sample characterized by XPS. b) Optical photographs of growth ampoules and the synthesized MoS_2 crystals. Not all the photographs are shown here. c) Schematic illustration depicting the onset of crystal nucleation for four representative samples (1-4), explaining the reason for the variation of Nb concentration in the bulk MoS_2 crystal.

S4. ToF-SIMS spectra

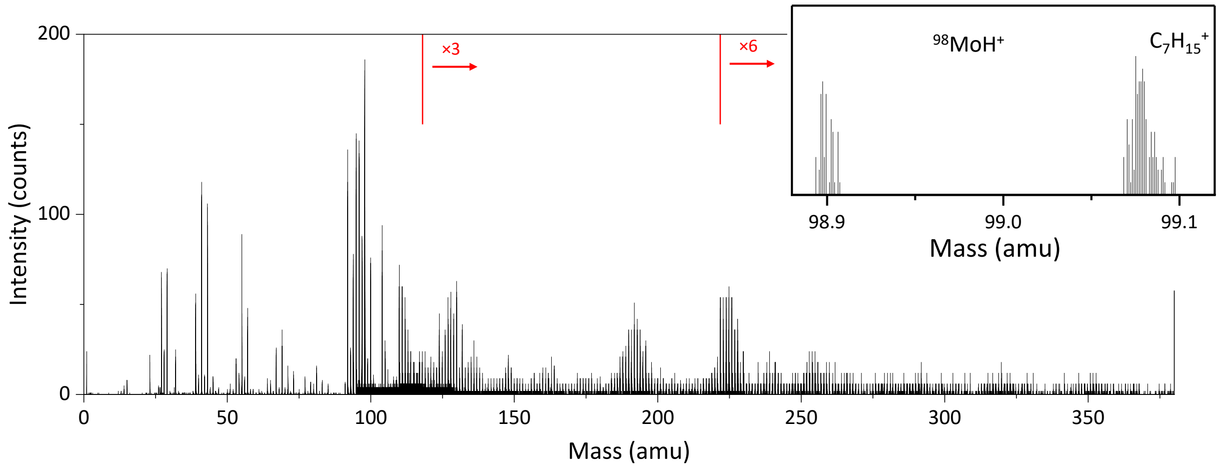

Figure S4. Full-range ToF-SIMS spectra acquired for the Nb-MoS_2 bulk sample, showing the distribution of secondary ions over a broad mass-to-charge (m/z) range from 0 amu to 300 amu. High mass resolution spectra in a selected region highlight the peaks corresponding to $^{98}MoH^+$ and $C_7H_{15}^+$, demonstrating the instrument's capability to resolve closely spaced peaks and distinguish between ions of similar m/z values.

S5. The difference of PL spectrum in pristine monolayer MoS_2 between the transfer methods

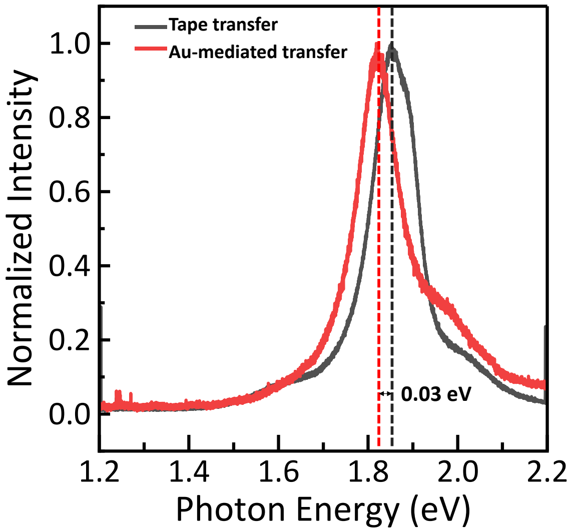

Figure S5. Comparison of room temperature PL spectra of pristine monolayer MoS_2 prepared by tape exfoliation and by the Au-mediated transfer method. Peak intensities are normalized. The redshift associated with the difference in preparations is at most a few tens of meV. Because all PL data in this work were acquired on samples prepared by the Au-mediated transfer method, the calibration curve (**Fig. 6**d) should be used with caution for tape exfoliated samples.

S6. Nb-MoS_2 bulk crystal for depth profiling

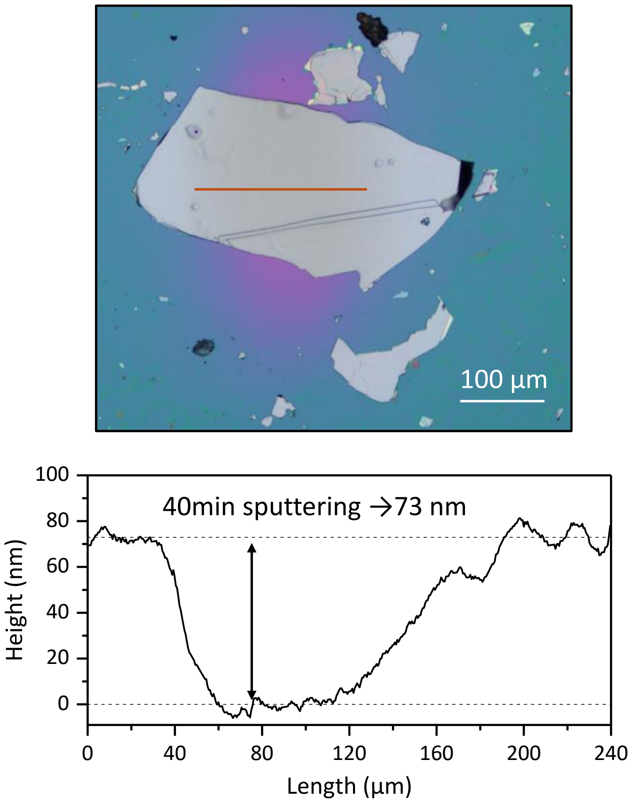

Figure S6. Optical photographs of the Nb-MoS_2 crystal after depth profiling. The sputtering rate was estimated to be ~1.8 nm per minute, based on laser profilometry measurements of crater depth along the red line in the photograph.

S7. Comparing doping methods for TMDs

Item/Methods	CVT[1]	CVD[1,2]	MOCVD[1,2]	Plasma substitution[3]	Plasma (non-substitutional)[3]	Molecular doping[4]
Substitutional	○ (Metal)	○ (Metal)	○ (Metal)	○ (Chalcogen)	×	×
Bulk	○	×	×	×	×	×
Uniformity	△(Our research)	△[2]	○[2]	○	○	○
Spatial selectivity	×	×	×	○	○	○
Post doping	×	×	×	○	○	○

Figure S7. Comparative table of doping methods for TMDs. This study shows that substitutional doping via CVT can exhibit variability, particularly at low dopant concentrations. Although CVT is an in-growth doping method and thus does not allow spatially selective doping, it remains the only available technique capable of achieving bulk substitutional doping with high crystal quality. Therefore, CVT-doped TMDs will continue to be essential for fundamental research and for enabling doping schemes critical for future device technologies.

Reference

[1] L. Loh, Z. Zhang, M. Bosman, G. Eda, *Nano Res*. **2021**, *14*, 1668.
[2] T. Kang, T. W. Tang, B. Pan, H. Liu, K. Zhang, Z. Luo, *ACS Mater. Au* **2022**, *2*, 665.
[3] S. Sovizi, S. Angizi, S. A. A. Alem, R. Goodarzi, M. R. Rahmani Taji Boyuk, H. Ghanbari, R. Szoszkiewicz, A. Simchi, P. Kruse, *Chem. Rev*. **2023**, *123*, 13869.
[4] Y. Wang, Y. Zheng, C. Han, W. Chen, *Nano Res*. **2021**, *14*, 1682.
